# Spontaneous dissection of the left gastric artery: a rare cause of abdominal pain

**DOI:** 10.1590/0100-3984.2017.0199

**Published:** 2019

**Authors:** Rômulo Florêncio Tristão Santos, Denise Maria Rissato Camilo, Thiago Alonso Domingos, Thiago Franchi Nunes, Edson Marchiori

**Affiliations:** 1 Universidade Federal de Mato Grosso do Sul (UFMS), Campo Grande, MS, Brazil.; 2 Universidade Anhanguera, Campo Grande, MS, Brazil.; 3 Universidade Federal do Rio de Janeiro (UFRJ), Rio de Janeiro, RJ, Brazil.

**Figure 1:**
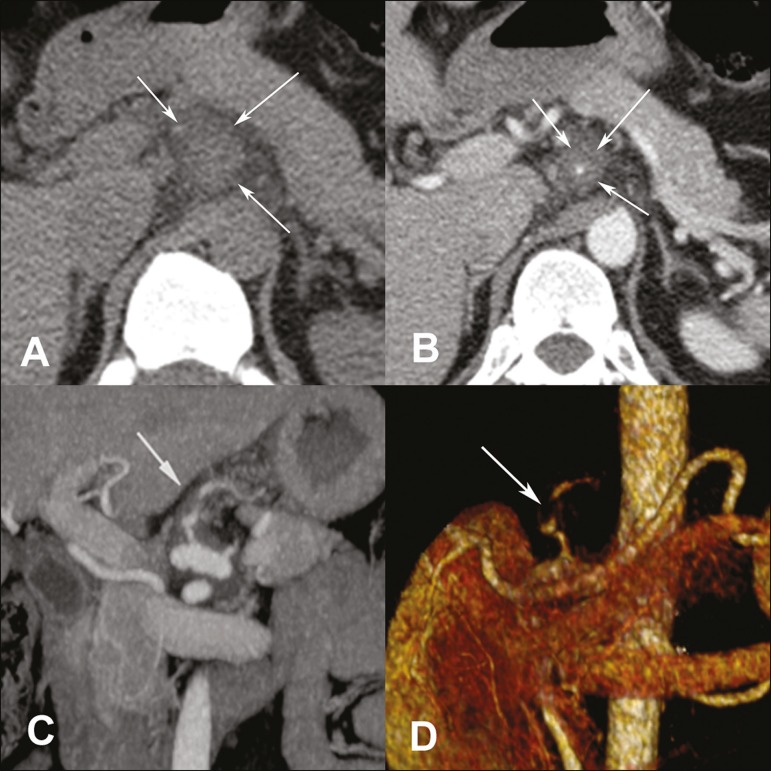
**A:** CT showing high attenuation (50 HU) of the tissue before contrast administration, consistent with hepatic thrombosis. **B:** Contrast-enhanced axial CT showing diffuse irregular thickening of the left gastric artery. **C,D:** 3D multiplanar reconstruction showing eccentric thickening suggestive of false lumen thrombosis.

Dear Editor,

A 44-year-old man was admitted to the emergency department with a 12-h history of severe epigastric pain. He reported no history of trauma or fall. Physical examination revealed a flaccid abdomen, pain on deep palpation of the epigastrium, and no signs of peritoneal irritation. The results of laboratory tests, including a complete blood count, together with the determination of the levels of amylase and transaminases, showed no relevant changes. Upper gastrointestinal endoscopy showed signs of mild non-erosive distal esophagitis and moderate erosive antral gastritis, as well as some sessile hyperplastic polyps in the gastric body. An abdominal ultrasound did not show any changes. Because of persistent pain, the patient underwent abdominal computed tomography (CT) angiography, which showed high attenuation of the tissue before contrast administration (Figure 1A). Contrast-enhanced axial CT showed diffuse irregular thickening of the left gastric artery (Figure 1B). Multiplanar reconstruction demonstrated eccentric thickening suggestive of false lumen thrombosis (Figure 1C). Three-dimensional (3D) reconstruction revealed diffuse irregular thickening of the left gastric artery (Figure 1D). These findings are consistent with a diagnosis of spontaneous dissection of the left gastric artery. No aneurysm formations or relevant anatomical variations were found in the evaluated arteries. A multidisciplinary group recommended conservative treatment (with anticoagulant/antiplatelet therapy and analgesics), hospital discharge, and outpatient follow-up.

Spontaneous dissection of a splanchnic artery is a rare event. Although several possible causes, including fibromuscular dysplasia, congenital connective tissue disorders, cystic medial necrosis, trauma, and hypertension, have been proposed, no strong association has yet been established^(^^1^^,^^2^^)^. Dissection of the superior mesenteric artery has been the most often described, although its incidence is estimated at only approximately 0.06%. To our knowledge, there has been only one reported case of isolated left gastric artery dissection without aneurysm formation^(^^3^^)^.

Acute abdomen has been the subject of recent publications in the radiology literature of Brazil^(^^4^^-^^8^^)^. CT angiography is the modality of choice for diagnosing cases with clinical suspicion of vascular pain. It is a rapid, noninvasive method that clearly shows vascular changes and possible anatomical variations^(^^9^^)^. New 3D reconstruction techniques help evaluate the extent of vascular involvement and improve the definition of a morphological pattern. Although considered a pathognomonic finding of spontaneous dissection, an intimal flap is not always clearly visualized in the images. Therefore, the diagnosis of dissection in such cases depends on a finding of false lumen thrombosis or eccentric mural thrombi.

There are several available treatment approaches to spontaneous dissection of a splanchnic artery, including conservative therapy with anticoagulation and blood pressure control; percutaneous endovascular interventions such as stent placement, embolotherapy, and intralesional thrombolytic therapy; and surgical interventions such as artery ligation, endoaneurysmorrhaphy, resection by laparotomy, and aortomesenteric bypass. Conservative management with anticoagulation is recommended as the first-line therapy. Endovascular treatment is indicated in cases of progression of the dissection, luminal thrombosis, increasing aneurysmal dilatation of the artery, or persistent symptoms despite anticoagulation. Emergency laparotomy with surgical repair should be performed in patients with suspicion of low blood flow and bowel necrosis or ruptured artery^(^^10^^-^^13^^)^.
